# Association between arterial health and cognition in adolescents: The PANIC study

**DOI:** 10.14814/phy2.16024

**Published:** 2024-05-02

**Authors:** Petri Jalanko, Bert Bond, Jari A. Laukkanen, Soren Brage, Ulf Ekelund, Tomi Laitinen, Sara Määttä, Mika Kähönen, Eero A. Haapala, Timo A. Lakka

**Affiliations:** ^1^ Faculty of Sport and Health Sciences University of Jyväskylä Jyväskylä Finland; ^2^ Helsinki Clinic for Sports and Exercise Medicine Foundation for Sports and Exercise Medicine Helsinki Finland; ^3^ Children's Health and Exercise Research Centre, Sport and Health Science, College of Life and Environmental Sciences University of Exeter Exeter UK; ^4^ Department of Medicine, Institute of Clinical Medicine University of Eastern Finland Kuopio Finland; ^5^ Department of Medicine Central Finland Health Care District Hospital District Jyväskylä Finland; ^6^ MRC Epidemiology Unit University of Cambridge School of Clinical Medicine Cambridge UK; ^7^ Department of Sports Medicine Norwegian School of Sport Sciences Oslo Norway; ^8^ Department of Clinical Physiology and Nuclear Imaging University of Eastern Finland and Kuopio University Hospital Kuopio Finland; ^9^ Department of Clinical Neurophysiology Kuopio University Hospital Kuopio Finland; ^10^ Department of Clinical Physiology Tampere University Hospital and Faculty of Medicine and Health Technology, Tampere University Tampere Finland; ^11^ Institute of Biomedicine, School of Medicine University of Eastern Finland Kuopio Finland; ^12^ Foundation for Research in Health Exercise and Nutrition Kuopio Research Institute of Exercise Medicine Kuopio Finland

**Keywords:** arterial stiffness, blood pressure, carotid intima‐media thickness, physical activity, sedentary time

## Abstract

We investigated the associations of the measures of arterial health with cognition in adolescents and whether physical activity (PA) or sedentary time (ST) confounds these associations. One hundred sixteen adolescents (71 boys) aged 15.9 ± 0.4 participated in the study. PA and ST were assessed using a combined accelerometer/heart rate monitor. Overall cognition was computed from the results of psychomotor function, attention, working memory, and paired‐associate learning tests. Pulse wave velocity was measured by impedance cardiography, carotid intima‐media thickness, and carotid artery distensibility by carotid ultrasonography. Systolic and diastolic blood pressure (SBP and DBP) were measured using an aneroid sphygmomanometer. SBP was inversely associated with overall cognition (standardized regression coefficient [*β*] = −0.216, 95% confidence interval (CI) −0.406 to −0.027, *p* = 0.025). Pulse wave velocity (*β* = −0.199, 95% CI −0.382 to −0.017, *p* = 0.033) was inversely associated with working memory task accuracy. SBP was directly associated with reaction time in the attention (*β* = 0.256, 95% CI 0.069 to 0.443, *p* = 0.008) and errors in the paired‐associate learning tasks (*β* = 0.308, 95% CI 0.126 to 0.489, *p* = 0.001). Blood pressure was inversely associated with overall cognition. PA or ST did not confound the associations. Results suggest that preventing high blood pressure is important for promoting cognition in adolescents.

## 1 INTRODUCTION

Arterial stiffness, elevated blood pressure, and measures of preclinical atherosclerosis, such as increased carotid intima‐media thickness, possibly leading to brain white matter microstructural alterations and weakened cerebral small vessel function, have been associated with poorer cognition and increased risk of cognitive decline in adults (Alvarez‐Bueno et al., 2020; Lamballais et al., 2018; Singer et al., 2014; Zeki Al Hazzouri et al., 2015; Zeki Al Hazzouri & Yaffe, 2014). In adolescents, evidence suggests that physical activity (PA) has a positive influence on arterial health and cognition, while high levels of sedentary time (ST) can have a negative impact on these outcomes (Esteban‐Cornejo et al., 2015; Herting & Chu, 2017; Hopkins et al., 2009; Saunders et al., 2014; Tremblay et al., 2011). However, little is known about the association between arterial health and cognition in adolescents. This research gap is striking because adolescence is a critical period for cognitive and brain development (Steinberg, 2005). Moreover, previous studies have not investigated whether PA or ST are key considerations in these associations.

Few available studies suggest weak, if any, associations between arterial stiffness and cognition in children and adolescents (Lamballais et al., 2018; Skog et al., 2020). In contrast, Vogrin et al. (2017) found a negative correlation of augmentation index, an indicator of arterial stiffness and peripheral tone, and central systolic artery pressure to academic performance among youth aged 11–16 years. Nevertheless, there is a lack of studies investigating the associations between arterial stiffness, cognition, PA, and ST in adolescents and exploring sex differences in these associations. Understanding this potential influence of sex is important, given that the rate of cognitive and brain maturation, arterial stiffness, and PA differ between girls and boys, which may influence the associations between arterial health and cognition. Previous studies on the association between arterial health and cognition in girls and boys have provided mixed results. Accordingly, Vogrin et al. (2017) found a negative correlation between augmentation index and academic performance in boys and a negative correlation of central systolic artery pressure with academic performance in girls, but they did not consider any confounding factors in their analyses (Vogrin et al., 2017). Moreover, a recent study in older adults reported that arterial stiffness was more strongly associated with cognitive impairment in men than women (Dao et al., 2022). While such differences may exist in adolescents, to the best of our knowledge, there are no previous studies on the associations between the measures of arterial stiffness and preclinical atherosclerosis, such as carotid intima‐media thickness and carotid artery distensibility with cognition in adolescent boys and girls. While increased PA and decreased ST can potentially improve cognition and arterial health (Esteban‐Cornejo et al., 2015; Herting & Chu, 2017; Hopkins et al., 2009), no studies have investigated whether PA of different intensities or ST confounds the association between arterial stiffness and cognition in adolescents.

Therefore, this study has two primary aims. First, we investigated the associations of arterial health quantified as the measures of arterial stiffness, carotid intima‐media thickness, and blood pressure with cognition in adolescents and whether PA or ST are confounding factors in these associations. Second, we explored sex differences in the associations between the measures of arterial health and cognition and whether PA or ST confounds the associations between the measures of arterial health and cognition differently in boys and girls. We hypothesized that higher arterial stiffness and blood pressure are associated with poorer cognition in adolescents, PA attenuates these associations, and that the associations of arterial stiffness, carotid intima‐media thickness, and blood pressure with cognition are stronger in boys than in girls.

## 2 METHODS

### 2.1 Study design and participants

This study utilizes cross‐sectional data from the 8‐year follow‐up assessments of the Physical Activity and Nutrition in Children (PANIC) study (Eloranta et al., 2011), which is a controlled lifestyle intervention study to investigate the effects of a combined physical activity and diet intervention on cardiometabolic risk factors in a population sample of children from the city of Kuopio, Finland.

During the first 2 years, the intervention included six physical activity and dietary counseling sessions with specified topics of physical activity, sedentary behavior, and diet. The intervention group was given fact sheets on these topics, verbal and written information on possibilities to exercise in community, and some exercise equipment and free admissions for indoor sports. The children were encouraged to participate in after‐school exercise clubs. After the first 2 years, the intervention included one counseling session per year. The control group received advice on health improving physical activity and diet but no active intervention. The study continues as a follow‐up study.

The Research Ethics Committee of the Hospital District of Northern Savo approved the study protocol in 2006 (Statement 69/2006) and 2015 (Statement 422/2015). Written informed consent was acquired from the parent or caregiver of each child, and every child provided assent to participation. The PANIC study has been carried out following the principles of the Declaration of Helsinki, as revised in 2008. The funding sources had no role in collecting, analyzing, or interpreting the data or the publication's approval or disapproval.

We invited 736 children 6–9 years of age who started the first grade in 16 primary schools of Kuopio in 2007–2009. Altogether 512 children (248 girls, 264 boys), who accounted for 70% of those invited, participated in the baseline examinations in 2007–2009. The participants did not differ in age, sex, or body mass index standard deviation score from all children who started the first grade in the city of Kuopio in 2007–2009. We excluded six children from the study at baseline because of physical disabilities that could hamper participation in the intervention or no time or motivation to attend the study. We also excluded two children whose parents later withdrew their permission to use the data of their children.

Finally, 277 (63%) adolescents who had participated in the 2‐year follow‐up attended the 8‐year follow‐up. A total of 161 adolescents were excluded from the analyses because they had no valid data on arterial health, cognition, or PA and ST. Therefore, a total of 116 adolescents (71 boys, 45 girls) aged 15.9 ± 0.4 years from the 8‐year follow‐up examinations were used for the present analyses. Descriptive characteristics, measures of arterial stiffness, carotid intima‐media thickness, blood pressure, and cognition did not differ between the included and excluded participants (*p* = 0.055–0.821). A total of 75 participants (47 boys; 28 girls) were included in the intervention group and 41 (24 boys; 17 girls) in the control group. Of the 116 participants, 5 (4 boys, 1 girl) had no data on pubertal status. However, we included these participants in the analyses because pubertal status was used only as a covariate. We replaced the missing data on pubertal status by multiple imputation using 10 imputed datasets.

### 2.2 Measurement of body size and pubertal status

Two repeated measurements of body mass were carried out by the InBody® 720 bioelectrical impedance device (Biospace, Seoul, South Korea) to an accuracy of 0.1 kg. The mean of these two values was used in the analyses. Body stature was measured three times the adolescents standing in the Frankfurt plane without shoes using a wall‐mounted stadiometer to an accuracy of 0.1 cm. The mean of the nearest two values was used in the analyses. BMI was calculated by dividing body mass (kg) with stature (m^2^), and the body mass index standard deviation score was calculated using the national references (Saari et al., 2011). Body fat percentage was measured using the Lunar® dual‐energy X‐ray absorptiometry device (GE Medical Systems, Madison, WI, USA). A research physician assessed pubertal status using the five‐stage scale described by Tanner. We used testicular volume assessed by an orchidometer to assess pubertal status in boys and breast development to assess pubertal status in girls (Tanner, 1962).

### 2.3 Measurement of PA and ST

PA was assessed using a combined heart rate and body movement sensor Actiheart® (CamNtech Ltd., Papworth, UK) for a minimum of four consecutive days and analyzed in 60 s epochs (Leppänen et al., 2022). The combined heart rate and movement sensor was attached to the child's chest with two standard electrocardiographic electrodes (Bio Protech Inc., Donghwa‐ri, South Korea). The children were asked to wear the monitor continuously, including sleep and water‐based activities. Heart rate data were cleaned and individually calibrated using parameters obtained from the maximal cycle exercise test and were combined with movement sensor data to derive PA energy expenditure. Instantaneous PA energy expenditure was estimated using branched equation modeling and expressed as time spent at intensity levels of standard metabolic equivalents (METs), one MET corresponding to 71.2 J/min/kg, in minutes per day (Leppänen et al., 2022). In the current analyses, VPA (vigorous‐intensity physical activity) was defined as PAs at ≥7 METs, MVPA (moderate to vigorous intensity physical activity) at ≥4 METs, MPA (moderate intensity physical activity) at >4.0 and ≤7.0 METs and ST at ≤1.5 standard METs, sleep excluded (Väistö et al., 2019). PA data were accepted as a valid day if there was a minimum of 48 h of activity recording in weekday and weekend day hours that included at least 12 h from morning (3–9 am), noon (9 am–3 pm), afternoon (3–9 pm), and night (9 pm–3 am) to avoid potential bias from over‐representing specific times and activities of the days.

### 2.4 Measurement of pulse wave velocity

Pulse wave velocity was measured with the Circmon® B202 impedance cardiography device (JR Medical Ltd, Saku Vald, 419 Estonia). The participants were asked to rest for 15 min in a supine position before the measurement. Next, current electrodes were placed on the distal parts of the extremities, slightly proximal to the wrists and the ankles. Voltage electrodes were placed proximal to the current electrodes, with a distance of 5 cm between the centers of the electrodes. The distal impedance plethysmogram was recorded from a popliteal artery at the knee joint level. The active electrode was placed on the lateral side of the knee joint, and the reference electrode on the calf. The distance between the electrodes was about 20 cm. Alternating electrical current was applied to current electrodes and change in whole‐body impedance was measured from voltage electrodes. The CircMon software estimates the foot of the impedance cardiography signal that coincides with pulse transmission in the aortic arch and the foot of the impedance plethysmogram signal that coincides with pulse transmission in the popliteal artery. Utilizing the measured pulse transit time (Δ*t*) and assessed distance (*L*) between these two sites, the CircMon software calculates pulse wave velocity using the equation: pulse wave velocity (m/s) = *L*/Δ*t* (Koivistoinen et al., 2011).

### 2.5 Measurement of carotid intima‐media thickness and carotid artery distensibility

For the assessment of carotid intima‐media thickness and elasticity of the left common carotid artery, carotid ultrasound imaging was performed utilizing the Acuson Sequoia 512 Ultrasound Mainframe® (Acuson, Mountain View, CA, USA) with a 14.0 MHz linear array transducer using a standardized protocol (Raitakari et al., 2003). The sonographers analyzed the ultrasound scans offline from the digitally stored images. Three measurements of the far wall at end‐diastole were taken to derive maximal carotid intima‐media thickness. For the assessments of carotid artery elasticity, the diameter of the common carotid artery at end‐diastole and end‐systole was measured at least twice. In addition, the sonographer measured SBP and diastolic blood pressure (DBP) from the brachial artery just before and directly after the ultrasound scans. The means of the end‐diastolic and end‐systolic diameters, as well as SBP and DBP values, were used to calculate arterial elasticity indices. Carotid artery distensibility was calculated as ([systolic diameter − diastolic diameter]/diastolic diameter)/(SBP − DBP).

### 2.6 Measurement of SBP and DBP

A research nurse measured SBP and DBP from the right arm using the Heine Gamma® G7 aneroid sphygmomanometer (Heine Optotechnik, Herrsching, Germany) to an accuracy of 2 mmHg (Lintu et al., 2014). The measurement protocol included a 5‐min rest and then three measurements in the sitting position at 2‐min intervals. The mean of all three values was used as SBP and DBP.

### 2.7 Assessment of cognition

CogState test battery was used to assess psychomotor function using the Detection Test, attention using the Identification Test, reaction time in working memory task using the One Back Task, working memory accuracy using the Two Back Task, and paired‐associate learning using the Continuous Paired Associate Learning Task (CogState Ltd, Melbourne, Australia) (see supplementary material for details) (Nyaradi et al., 2014). An overall cognition was calculated by taking the average of the standardized scores across all CogState tests (Table 2). For the tests for which a lower score indicated better performance, the score was reversed (multiplied by −1) so that all outcome variables were in a uniform direction. Thus, a higher score indicated better performance. The associations of pulse wave velocity, carotid artery distensibility, carotid intima‐media thickness, blood pressure, PA, and ST with individual measures of cognitive functions are presented in the supplementary table.

The construct validity of the CogState test battery has been demonstrated in a large group of healthy adults and children (Maruff et al., 2009; Mollica et al., 2005). The test can be used in different cultures since there are only minimal language requirements to undertake the test battery (Cairney et al., 2007).

### 2.8 Statistical analyses

All analyses were carried out using the SPSS statistical analysis software, version 23.0 (IBM Corp., Armonk, NY, USA). The normality of the variables' distributions was tested with the Kolmogorov–Smirnov test and visually from histograms. Differences in descriptive characteristics, pulse wave velocity, carotid artery distensibility, carotid intima‐media thickness, blood pressure, PA and ST between sexes were analyzed by the Student *t*‐test, the Mann Whitney *U*‐test or the Chi‐square test. Associations of pulse wave velocity, carotid artery distensibility, carotid intima‐media thickness, blood pressure, PA and ST with cognition were investigated using linear regression analyses adjusted for age, sex, parental education and additionally body fat percentage, pubertal status and the study group. The data were adjusted for parental education as it has been associated directly with cognition (Cabrera et al., 2007) and PA (Muñoz‐Galiano et al., 2020). Age, sex, and parental education were entered into the regression model at the first step, and the pulse wave velocity, carotid artery distensibility, carotid intima‐media thickness, blood pressure, PA and ST were entered separately into the model at the second step. The data were corrected for multiple comparisons using the Benjamini–Hochberg false discovery rate (FDR) with an FDR value of 0.2 (FDR_0.2_). If the associations between arterial stiffness, carotid intima‐media thickness or blood pressure with cognition were statistically significant after adjustment for age, sex, parental education and additionally for body fat percentage, pubertal status and the study group, the data were further adjusted for TPA, MPA MVPA, VPA or ST, which were entered to models separately. Analyses by sex were conducted similarly, except for not using sex as a covariate. Standardized regression coefficients and their 95% confidence intervals with the corresponding *p*‐values were reported for each factor. Differences and associations with *p*‐values ≤0.05 were considered statistically significant. Finally, we investigated whether sex modified the associations of pulse wave velocity, carotid artery distensibility, carotid intima‐media thickness, blood pressure, PA, and ST with cognition using general linear models. We estimated statistical power for our analyses using the G*Power software. Altogether, 84 observations were needed to observe the correlation of 0.3 at the power of 0.80 when the statistical significance level was set at *p* < 0.05. Moreover, a correlation coefficient needed to reveal statistical significance at the level of *p* < 0.05 was 0.33 in boys and 0.40 in girls.

## 3 RESULTS

### 3.1 Characteristics of participants

Boys were taller, heavier, and had lower body fat percentage than girls (Table 1). Boys had higher resting SBP than girls. However, girls had higher carotid artery distensibility than boys. Moreover, boys accumulated more MVPA and VPA than girls.

**TABLE 1:** Descriptive characteristics of participants.

	All (*n* = 116)	Boys (*n* = 71)	Girls (*n* = 45)	*p*‐value
Age (years)	15.9 ± 0.4	15.9 ± 0.4	15.8 ± 0.4	0.595
Body mass (kg)	64.0 ± 14.4	68.2 ± 15.9	57.3 ± 8.0	**<0.001**
Body stature (cm)	172.8 ± 8.7	177.0 ± 7.9	166.4 ± 5.3	**<0.001**
Body mass index standard deviation score	0.00 ± 1.0	0.1 ± 1.1	−0.1 ± 0.8	0.566
Body fat percentage (%)	22.1 ± 10.4	18.6 ± 10.8	29.7 ± 6.6	**<0.001**
Pubertal status, *n* (%)^a^				0.060
Stage 3	10 (9)	9 (13.4)	1 (2.3)	
Stage 4	63 (56.8)	38 (56.7)	25 (56.8)	
Stage 5	38 (34.2)	20 (29.9)	18 (40.9)	
Systolic blood pressure (mmHg)	114 ± 12	116 ± 12	109 ± 10	**<0.001**
Diastolic blood pressure (mmHg)	68 ± 10	69 ± 10	67 ± 10	0.382
Blood pressure stages *n* (%)				
Normal (BP <120/<80 mmHg)	82 (70.7)	40 (56.3)	42 (93.3)	
Pre‐hypertensive (BP 120/<80–129/<80 mmHg)	21 (18.1)	21 (29.6)	0	
Stage 1 HTN (BP 130/80–139/89 mm Hg)	12 (10.3)	9 (12.7)	3 (6.7)	
Stage 2 HTN (BP >140/90 mmHg)	1 (0.9)	1 (1.4)	0	
Average of right and left leg pulse wave velocity (m/s)	5.9 ± 0.7	5.8 ± 0.6	5.9 ± 0.8	0.552
Carotid Intima‐media thickness (mm)	0.45 ± 0.06	0.46 ± 0.07	0.43 ± 0.05	0.123
Carotid artery distensibility (%/10 mmHg)	2.6 ± 0.7	2.5 ± 0.7	2.8 ± 0.6	**0.026**
Sedentary time (min/day)	605.8 ± 141.9	602.8 ± 147.0	610.5 ± 134.8	0.757
Moderate physical activity (min/day)	34.1 ± 25.7	37.1 ± 27.2	29.3 ± 22.5	0.111
Moderate‐to‐vigorous physical activity (min/day)	44.6 ± 36.4	50.6 ± 39.5	35.2 ± 29.0	**0.019**
Vigorous physical activity (min/day)	10.5 ± 14.3	13.5 ± 16.2	5.9 ± 8.8	**0.008**
Total physical activity (min/day)	367.6 ± 136.9	376.3 ± 143.2	354.1 ± 126.7	0.446
Parental education (%)				0.137
Vocational school or less	15.1	14.3	16.3	
Polytechnic	43.4	36.5	53.5	
University degree	41.5	49.2	30.2	

*Note*: The data are means (SDs) and corresponding *p*‐values from the *t*‐test for independent samples for continuous variables with normal distributions, from the Mann–Whitney *U*‐test for continuous variables with skewed distributions and the chi‐square test for categorical variables. *p*‐values indicating statistically significant differences are bolded.

Abbreviation: HTN, hypertension.

^a^ *n* = 111 (boys 67; girls 44).

### 3.2 Associations of arterial stiffness, carotid intima‐media thickness, blood pressure, PA, and ST with overall cognition

**FIGURE 1:**
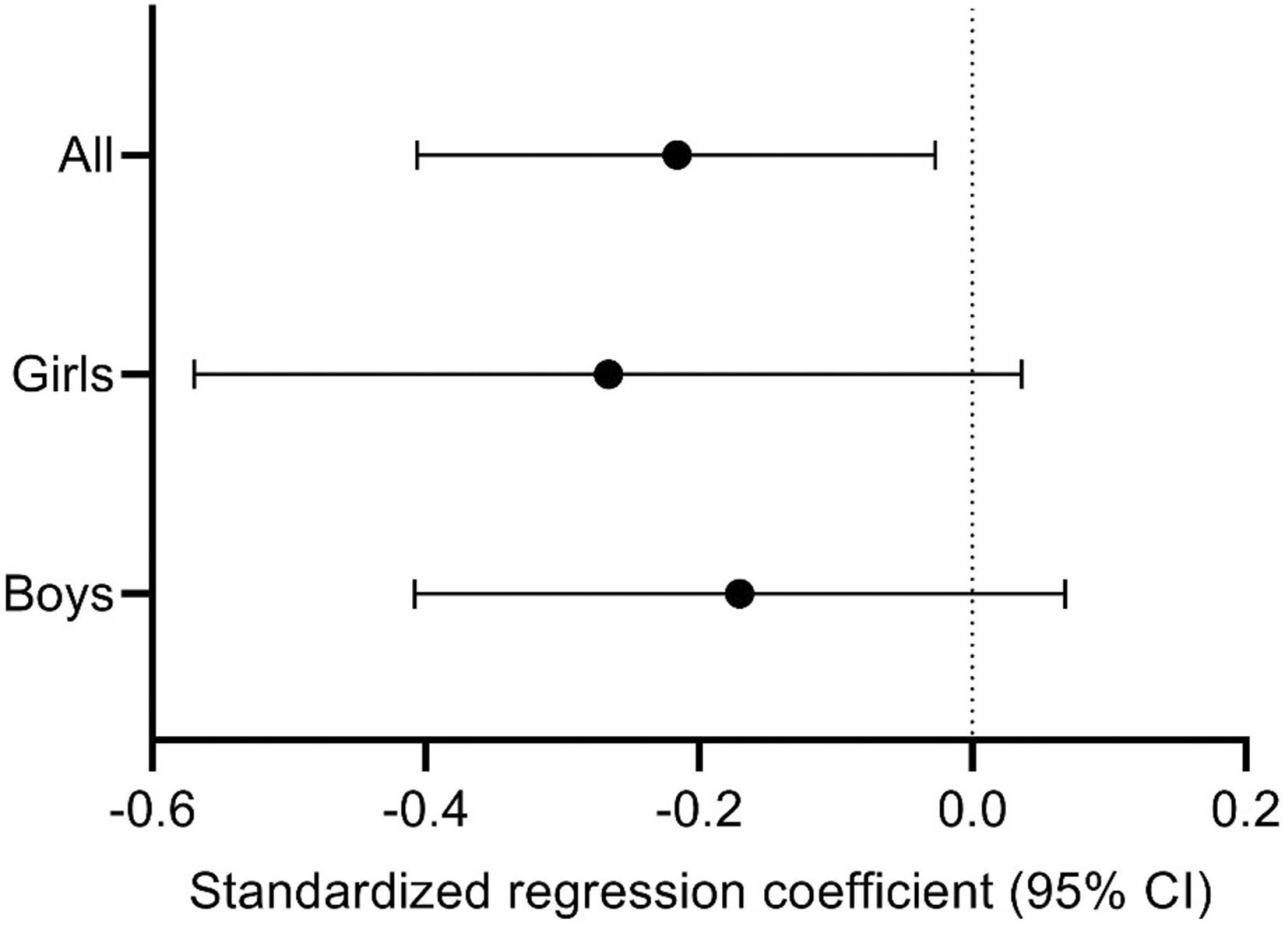
Associations between systolic blood pressure and overall cognition. All: *β* = −0.216, 95% CI −0.406 to −0.027; girls: *β* = −0.266, 95% CI −0.569 to 0.036; boys: *β* = −0.170, 95% CI −0.408 to 0.068. Data are standardized regression coefficients with their 95% confidence intervals adjusted for age, sex and parental education (all) or age and parental education (girls, boys). CI, confidence interval.

Higher SBP was associated with poorer overall cognition after adjustment for age, sex, and parental education (Table 2; Figure 1). The association remained after FDR_0.2_ correction. The association remained materially unchanged after further adjustment for body fat percentage, pubertal status and TPA, MPA, MVPA, VPA, or ST. Additional adjustment for the study group had no effect on the association. Other measures of arterial health were not associated with overall cognition.

**TABLE 2:** Associations of pulse wave velocity, carotid artery distensibility, carotid intima‐media thickness, blood pressure, PA, and ST with cognition.

	Overall cognition	
	Β (95% CI)	*p*
Pulse wave velocity (m/s)	0.080 (−0.106, 0.266)	0.397
Carotid Intima‐media thickness (mm)	−0.067 (−0.254, 0.119)	0.476
Carotid artery distensibility (%/10 mmHg)	0.119 (−0.074, 0.312)	0.224
Systolic blood pressure (mmHg)	**−0.216 (−0.406, −0.027)**	0.025*
Diastolic blood pressure (mmHg)	−0.110 (−0.297, 0.077)	0.246
Total physical activity (min/day)	0.047 (−0.138, 0.231)	0.617
Moderate physical activity (min/day)	0.078 (−0.108, 0.263)	0.408
Moderate‐to‐vigorous physical activity (min/day)	0.085 (−0.102, 0.272)	0.368
Vigorous physical activity (min/day)	0.078 (−0.112, 0.267)	0.419
Sedentary time (min/day)	−0.020 (−0.204, 164)	0.827

*Note*: The data are standardized regression coefficients with corresponding 95% confidence intervals (95% CI) and *p*‐values for each factor from linear regression analyses adjusted for age, sex, and parental education. Statistically significant associations and corresponding *p*‐values are bolded. *P‐value <0.05. Moderate physical activity (at >4.0 and ≤7.0 METs); Moderate‐to‐vigorous physical activity (>4.0 standard METs); Vigorous physical activity (>7.0 standard METs); Sedentary time (≤1.5 standard METs, sleep excluded). An overall cognition was calculated by taking the average of the standardized scores across all CogState tests: Detection Test, Identification Task, One Back Task, Two Back Task, and Continuous Paired Associate Learning Task. For the tests for which a lower score indicated better performance, the score was reversed (multiplied by −1) so that all outcome variables were in a uniform direction. Thus, a higher score indicated better performance.

Abbreviations: PA, physical activity; ST, sedentary time.

### 3.3 Associations of arterial stiffness, carotid intima‐media thickness, blood pressure, PA, and ST with overall cognition in boys (*N* = 71) and girls (*N* = 45)

Arterial stiffness, carotid intima‐media thickness, blood pressure, PA, and ST were not associated with overall cognition in the sex‐stratified analyses.

Associations of arterial stiffness, carotid intima‐media thickness, blood pressure, PA, and ST with individual measures of cognition and sex‐stratified analyses are presented in the supplementary table and text.

## 4 DISCUSSION

We found that higher SBP was associated with poorer overall cognition in adolescents. In the analyses for individual measures of cognition, higher pulse wave velocity was associated with poorer working memory and higher SBP was associated with poorer attention and paired associate learning. Moreover, higher pulse wave velocity was associated with better attention and working memory and higher SBP was associated with poorer paired‐associate learning in boys. In girls, higher pulse wave velocity and DBP were associated with poorer working memory, and higher SBP was associated with poorer paired‐associated learning. Finally, we found that pubertal status in boys and body fat percentage in girls modified some of the associations between arterial health and cognition, suggesting that accounting for maturation and body fat percentage in such analyses is essential to quantify the independent role of arterial health in cognitive development in adolescents. As additional adjustments for pubertal status and body fat percentage influenced some associations, future studies investigating the associations between arterial health and cognition in adolescents must take pubertal status and body fat percentage into account.

We found negative associations between blood pressure and overall cognition, as well as several individual measures of cognitive functions. These results are consistent with previous research conducted in children and adolescents with hypertension, summarized in a review by Cha et al. (2012). Additionally, we observed inverse associations between pulse wave velocity and cognition, aligning with observations in adults (Singer et al., 2014). However, our findings contradict previous studies conducted in children and adolescents, which showed weak or no associations between pulse wave velocity and cognition (Lamballais et al., 2018; Skog et al., 2020; Vogrin et al., 2017). These results indicate that increased pulse wave velocity may have a more significant impact on cognition during adolescence than earlier in life. Furthermore, previous studies in children and adolescents (Skog et al., 2020; Vogrin et al., 2017) may have been underpowered to detect statistically significant associations due to small sample sizes. Moreover, to the best of our knowledge, we report here for the first time results on the associations between carotid intima‐media thickness and cognition, suggesting that higher thickness in the inner layers of the carotid artery is not associated with cognition in adolescents. While different measures of arterial stiffness and cognition used make it challenging to compare the results of studies, our findings suggest that increased blood pressure and arterial stiffness could impair cognition in children and adolescents. It is beyond the scope of our study to identify mechanisms that underlie these associations, but they could include structural changes in the brain, primarily white matter hyperintensities and cortical brain atrophy, or impaired cerebral small vessel function. Aortic stiffening and high blood pressure may impair cerebral small vessel function by abnormal flow pulsations into the brain microcirculation reducing cerebral blood flow, impairing cerebral autoregulation and increasing blood–brain barrier permeability (Singer et al., 2014; Walker et al., 2017). Although the hypothesis of mechanisms is intriguing, it is merely speculative as we did not measure brain structures.

In one of the few studies on the sex‐stratified associations between the measures of arterial health and cognitive functions, Vogrin et al. (2017) reported a negative correlation between SBP and academic performance in girls, but not in boys, in a sample of youth aged 11–16. Similarly, we found that higher SBP and DBP were inversely associated with a larger number of individual measures of cognitive functions in girls than in boys. Thus, elevated blood pressure may be a more important determinant of cognition in girls than in boys across childhood and adolescence. Vogrin et al. (2017) also found that the augmentation index (%), an indicator of peripheral arterial tone and arterial stiffness, but not pulse wave velocity, negatively correlated to academic performance in boys but not girls. In contrast, we found that higher pulse wave velocity, indicating higher total body arterial stiffness, was associated with better attention and working memory in boys, but not in girls. Differences in sample characteristics, such as age, maturation status, and sample size, may explain these contrasting findings. In addition, we used impedance cardiography to assess aortic‐popliteal artery pulse wave velocity using two measurement sites. In contrast, Vogrin et al. (2017) used an oscillometric device to estimate pulse wave velocity from a single brachial artery site recording. As two measurement site methods are more appropriate and valid in assessing pulse wave velocity than single site methods (Ferreira & Gill, 2023), a more precise assessment of pulse wave velocity may have driven a stronger association between arterial stiffness and cognition in our study. Nevertheless, more research on the associations of arterial health assessed from different arterial segments with cognition in adolescents is warranted. The sex differences in the association between blood pressure and cognition might be partly explained by the intrinsic genetic and hormonal differences, as both sex hormones, testosterone and estrogen, have been found to increase arterial compliance (Ahimastos et al., 2003; DuPont et al., 2019).

To the best of our knowledge, this is the first study to investigate whether PA and ST confound the associations of arterial stiffness and blood pressure with cognition in adolescents. The results suggest that PA and ST do not confound the associations, which is explained by our study's weak associations between PA, ST, and cognition. While a systematic review (Esteban‐Cornejo et al., 2015) suggested a positive association between PA and cognition in adolescents, only one study in their systematic review used a device‐assessed measure of physical activity, which might explain the contrary findings to our study.

The strengths of the present study are a relatively large sample of adolescents and valid and reproducible measures of arterial health (Laurent et al., 2006), cognition, PA, and ST (Brage et al., 2005; Helmerhorst et al., 2012; Prince et al., 2008). However, we had to exclude a relatively large number of participants due to missing data. Nevertheless, the included adolescents did not differ in descriptive characteristics, measures of arterial stiffness, blood pressure, or cognition from those excluded from the analyses. Moreover, we investigated the associations between indices of large artery health, instead of cerebrovascular health, and cognition. However, while the evidence in adolescents is limited, large artery stiffness has been associated with impaired cerebral small vessel health in adults (Singer et al., 2014). Finally, the cross‐sectional nature of the study does not allow us to make conclusions about the causality or directionality of the associations.

In conclusion, higher blood pressure and arterial stiffness were related to poorer cognition in adolescents. There were sex differences in the associations of arterial health with measures of cognition, as the strength and direction of associations were sex‐specific. PA or ST did not confound the associations. Therefore, our results highlight the importance of preventing high blood pressure and arterial stiffening to promote cognitive and brain health in youth. Future randomized controlled trials with appropriate control groups and novel brain imaging are needed to investigate the mediating effects of arterial health and the moderating role of sex in the effects of PA and ST on cognition in adolescents.

## AUTHOR CONTRIBUTIONS

P.J., B.B., J.A.L., S.B., U.E., T.L., S.M., M.K., E.A.H., and T.A.L. contributed to the conception or design of the study and analysis or interpretation of the data. P.J. and E.A.H. drafted the manuscript, and B.B, J.A.L., S.B., U.E., T.L., S.M., M.K., and T.A.L. critically revised the manuscript. All authors gave final approval and agreed to be accountable for all aspects of the work, ensuring integrity and accuracy. The paper is original, and it or parts of it have not been published elsewhere.

## FUNDING INFORMATION

The author(s) disclosed receipt of the following financial support for the research, authorship, and/or publication of this article: The PANIC Study has financially been supported by the Juho Vainio Foundation, Ministry of Education and Culture of Finland, Ministry of Social Affairs and Health of Finland, Research Committee of the Kuopio University Hospital Catchment Area (State Research Funding), Finnish Innovation Fund Sitra, Social Insurance Institution of Finland, Finnish Cultural Foundation, Foundation for Pediatric Research, Diabetes Research Foundation in Finland, Finnish Foundation for Cardiovascular Research, Paavo Nurmi Foundation, Yrjö Jahnsson Foundation, and the city of Kuopio. Finnish Foundation for Cardiovascular Research, Urheiluopisto Foundation, Päivikki and Sakari Sohlberg Foundation, Yrjö Jahnssons Foundation, Aarne Koskelo Foundation, and Juho Vainio Foundation financially supported Petri Jalanko.

## CONFLICT OF INTEREST STATEMENT

The author(s) declared no potential conflicts of interest with respect to the research, authorship, and/or publication of this article.

## ETHICS STATEMENT

The Research Ethics Committee of the Hospital District of Northern Savo approved the study protocol in 2006 (Statement 69/2006) and 2015 (Statement 422/2015).

## PATIENT CONSENT STATEMENT

Written informed consent was acquired from the parent or caregiver of each child, and every child provided assent to participation.

## PERMISSION TO REPRODUCE MATERIAL FROM OTHER SOURCES

Materials from other sources were not included.

## Supporting information


Data S1:

## Data Availability

The data that support the findings of this study are available from the corresponding author, Jalanko P., upon reasonable request.
