# Supplementary material for: Association between arterial health and cognition in adolescents: The PANIC study
Source: Physiol Rep. 2024 May 2;12(9):e16024. doi: 10.14814/phy2.16024 (PMC11065692; doi:10.14814/phy2.16024)
Supplement: Supplementary file 1 — Data S1: [file PHY2-12-e16024-s001.zip › PHYSREP-2023-11-493-file002.doc]

|  | **Detection test**  **(Speed of performance)** | | **Identification test (Speed of performance)** | | **One Back Task (Speed of performance)** | | **Two Back Task (Speed of performance)** | | **Identification test (accuracy)** | | **One Back Task (accuracy)** | | **Two Back Task (accuracy)** | | **Continuous Paired Associate Learning test (errors)** | |
| --- | --- | --- | --- | --- | --- | --- | --- | --- | --- | --- | --- | --- | --- | --- | --- | --- |
|  | **β**  **(95% CI)** | **p** | **β**  **(95% CI)** | **p** | **β**  **(95% CI)** | **p** | **β**  **(95% CI)** | **p** | **β**  **(95% CI)** | **p** | **β**  **(95% CI)** | **p** | **β**  **(95% CI)** | **p** | **β**  **(95% CI)** | **p** |
| **Pulse wave velocity (m/s)** | -0.175  (-0.356,  0.007) | 0.059 | -0.144  (-0.329,  0.040) | 0.123 | -0.071  (-0.256,  0.113) | 0.447 | -0.134  (-0.317,  0.050) | 0.151 | -0.006  (-0.196,  0.184) | 0.952 | **-0.199**  **(-0.382,**  **-0.017)** | **0.033*** | -0.021  (-0.211,  0.169) | 0.829 | -0.045  (-0.228,  0.138) | 0.628 |
| **Carotid Intima-media thickness (mm)** | -0.026  (-0.211,  0.158) | 0.780 | -0.010  (-0.196,  0.176) | 0.915 | 0.147  (-0.036,  0.330) | 0.114 | 0.044  (-0.141,  0.229) | 0.637 | 0.021  (-0.169,  0.211) | 0.829 | -0.103  (-0.289,  0.082) | 0.272 | -0.080  (-0.270,  0.109) | 0.402 | -0.030  (-0.213,  0.154) | 0.748 |
| **Carotid artery distensibility (%/10 mmHg)** | -0.001  (-0.193,  0.191) | 0.994 | -0.102  (-0.295,  0.091) | 0.297 | -0.138  (-0.329,  0.053) | 0.135 | -0.137  (-0.328, 0.054) | 0.158 | -0.051  (-0.248,  0.147) | 0.612 | -0.054  (-0.248,  0.140) | 0.583 | 0.085  (-0.112,  0.282) | 0.397 | -0.154  (-0.342,  0.035) | 0.110 |
| **Systolic blood pressure (mmHg)** | 0.153  (-0.036,  0.343) | 0.112 | **0.256**  **(0.069, 0.443)** | **0.008**** | 0.081  (-0.110,  0.273) | 0.401 | 0.085  (-0.106,  0.277) | 0.378 | -0.076  (-0.272,  0.121) | 0.488 | 0.039  (-0.154,  0.232) | 0.690 | -0.009  (-0.206,  0.188) | 0.930 | **0.308**  **(0.126,**  **0.489)** | **0.001***** |
| **Diastolic blood pressure (mmHg)** | 0.007  (-0.178,  0.192) | 0.939 | 0.077  (-0.110, 0.263) | 0.417 | 0.079  (-0.107,  0.264) | 0.402 | 0.098  (-0.086,  0.283) | 0.294 | 0.092  (-0.098,  0.282) | 0.339 | -0.129  (-0.315,  0.057) | 0.172 | -0.085  (-0.275,  0.105) | 0.377 | 0.088  (-0.096,  0.271) | 0.345 |
| **Total physical activity (min/d)** | -0.077  (-0.259,  0.105) | 0.404 | -0.041  (-0.225,  0.143) | 0.657 | 0.077  (-0.105,  0.260) | 0.404 | 0.042  (-0.141,  0.225) | 0.648 | 0.018  (-0.170,  0.206) | 0.848 | 0.056  (-0.128,  0.241) | 0.547 | 0.130  (-0.057,  0.316) | 0.171 | 0.003  (-0.179,  0.184) | 0.976 |
| **Moderate physical activity (min/d)** | -0.038  (-0.222,  0.146) | 0.683 | -0.145  (-0.328,  0.039) | 0.121 | 0.053  (-0.131,  0.237) | 0.572 | -0.052  (-0.236,  0.133) | 0.580 | 0.115  (-0.074,  0.303) | 0.230 | -0.034  (-0.220,  0.152) | 0.715 | 0.073  (-0.116,  0.261) | 0.448 | 0.001  (-0.182,  0.184) | 0.992 |
| **Moderate-to-vigorous physical activity (min/d)** | -0.062  (-0.247,  0.124) | 0.511 | -0.170  (-0.354,  0.014) | 0.070 | 0.045  (-0.140,  0.231) | 0.630 | -0.015  (-0.201,  0.171) | 0.870 | 0.122  (-0.068,  0.312) | 0.205 | -0.038  (-0.225,  0.149) | 0.689 | 0.075  (-0.116,  0.265) | 0.438 | -0.006  (-0.190,  0.179) | 0.951 |
| **Vigorous physical activity (min/d)** | -0.090  (-0.277,  0.097) | 0.343 | -0.174  (-0.360,  0.013) | 0.068 | 0.020  (-0.168,  0.208) | 0.835 | 0.056  (-0.132,  0.245) | 0.554 | 0.105  (-0.088,  0.297) | 0.284 | -0.035  (-0.225,  0.155) | 0.715 | 0.060  (-0.133,  0.253) | 0.540 | -0.017  (-0.203,  0.170) | 0.860 |
| **Sedentary time (min/d)** | 0.103  (-0.078,  0.284) | 0.261 | 0.053  (-0.130,  0.236) | 0.568 | -0.122  (-0.303,  0.059) | 0.184 | -0.092  (-0.273,  0.090) | 0.320 | -0.003  (-0.191,  0.184) | 0.972 | -0.003  (-0.187,  0.181) | 0.973 | -0.131  (-0.317,  0.055) | 0.165 | 0.007  (-0.173,  0.188) | 0.935 |

**Supplementary Table**. Associations of pulse wave velocity, carotid artery distensibility, carotid intima-media thickness, blood pressure, PA and ST with individual measures of cognition

The data are standardised regression coefficients with corresponding 95% confidence intervals (95% CI) and P-values for each factor from linear regression analyses adjusted for age, sex and parental education. Statistically significant associations and corresponding *P*-values are bolded. Moderate physical activity (at > 4.0 and ≤ 7.0 METs); Moderate-to-vigorous physical activity (> 4.0 standard METs); Vigorous physical activity (> 7.0 standard METs); Sedentary time (≤ 1.5 standard METs, sleep excluded) Abbreviations: MET, metabolic equivalents
