# Supplementary material for: Association between arterial health and cognition in adolescents: The PANIC study
Source: Physiol Rep. 2024 May 2;12(9):e16024. doi: 10.14814/phy2.16024 (PMC11065692; doi:10.14814/phy2.16024)
Supplement: Supplementary file 1 — Data S1: [file PHY2-12-e16024-s001.zip › PHYSREP-2023-11-493-file003.doc]

*Associations of arterial stiffness, carotid intima-media thickness, blood pressure, PA and ST with* *individual measures of cognition*

A higher pulse wave velocity was associated with poorer accuracy in the One Back Task test after adjustment for age, sex, and parental education (Supplementary table). The association remained after FDR_0.2_ correction. However, the association did not remain statistically significant after further adjustment for body fat percentage (β=-0.174, 95 % CI -0.362 to 0.014, *P*=0.069). Additional adjustment for pubertal status and the study group had no effect on the association. Higher SBP was associated with a longer reaction time in the Identification Test, and a higher number of errors in the Continuous Paired Associate Learning Task after adjustment for age, sex and parental education. Both associations remained after FDR_0.2_ correction. Both associations remained materially unchanged after further adjustment for body fat percentage and TPA, MPA, MVPA, VPA, or ST. Additional adjustment for pubertal status and the study group had no effect on the associations.

*Associations of arterial stiffness, carotid intima-media thickness, blood pressure, PA and ST with individual measures of cognition in boys (N=71) and girls (N=45)*

A Higher pulse wave velocity was associated with shorter reaction times in the Identification Test in boys (β=-0.247, 95% CI -0.486 to -0.007, *P*=0.044), after adjustment for age and parental education. The association did not remain after FDR_0.2_ correction but remained statistically significant after further adjustment for body fat percentage. However, the association did not remain statistically significant after further adjustment for pubertal status (β=-0.209, 95% CI -0.460 to 0.041 *P*=0.102). Additional adjustment for study group had no effect on the association. Higher pulse wave velocity was associated with shorter reaction times in the Detection Test (β=-0.242, 95% CI -0.484 to -0.001, *P*=0.049) task in boys after adjustment for age and parental education. The association did not remain after FDR_0.2_ correction. The association remained statistically significant after further adjustment for body fat percentage but was no longer statistically significant after additional adjustment for pubertal status (β=-0.173, 95% CI -0.426 to 0.072, *P*=0.164). Additional adjustment for study group had no effect on the association. Higher pulse wave velocity was associated with a shorter reaction time in the One Back Task in boys (β=-0.262, 95% CI -0.499 to -0.024, *P*=0.031), but not in girls (β=0.130, 95% CI -0,170 to 0.430, *P*=0.385; *P*=0.041 for interaction. The association remained after FDR_0.2_ correction and remained statistically significant after further adjustment for body fat percentage, TPA, MPA, MVPA, VPA, or ST. Additional adjustment for pubertal status and study group had no effect on the association. Higher SBP was associated with more errors in the Continuous Paired Associate Learning Task in boys (β=0.286, 95% CI 0.052 to 0.521, *P*=0.017) and girls (β=0.335, 95 % CI 0.057 to 0.613, *P*=0.020, *P=*0.984 for interaction). The associations remained after FDR_0.2_ correction and remained statistically significant after further adjustment for body fat percentage, TPA, MPA, MVPA, VPA, or ST. Additional adjustment for pubertal status and study group had no effect on the association.

Higher pulse wave velocity was associated with poorer accuracy in One Back Task in girls (β=-0.329, 95 % CI -0.629 to -0.030, *P*=0.032). The association remained after FDR_0.2_ correction. However, the association did not remain statistically significant after additional adjustment for body fat percentage (β=-0.287, 95 % CI -0.610 to -0.036, *P*=0.080). Additional adjustment for pubertal status and study group had no effect on the association. Higher DBP was associated with poorer accuracy in One Back Task in girls (β=-0.318, 95 % CI -0.625 to -0.011, *P*=0.042). The association remained after FDR_0.2_ correction. However, the association did not remain statistically significant after additional adjustment for body fat percentage (β=-0.285, 95 % CI -0.597 to 0.027, *P*=0.073). Additional adjustment for pubertal status and study group had no effect on the association.

*Sex differences in the associations of PA, ST and arterial stiffness with cognition*

In addition to the sex difference mentioned above, the associations between carotid intima-media thickness and Detection Test (*P*=0.036 for interaction), pulse wave velocity and reaction times in One Back Task (*P*=0.041 for interaction), and ST and accuracy in One Back Task (*P*=0.048 for interaction) were different between boys and girls. However, these associations of arterial stiffness measures and ST with cognition by sex were not statistically significant.
