# Supplementary material for: Association between arterial health and cognition in adolescents: The PANIC study
Source: Physiol Rep. 2024 May 2;12(9):e16024. doi: 10.14814/phy2.16024 (PMC11065692; doi:10.14814/phy2.16024)
Supplement: Supplementary file 1 — Data S1: [file PHY2-12-e16024-s001.zip › PHYSREP-2023-11-493-file004.doc]

**Supplementary material**

*Assessment of cognition*

The psychomotor function was assessed with the Detection Test. The participants were asked to click a button as quickly as possible when a playing card flipped over on a computer screen during the test. The test result was based on the speed of performance. A lower score indicated better performance.

Attention was assessed by the Identification Task. The participants were asked to choose whether a revealed card was red or not during the task. The participants were required to press "yes" for the red card, and “no” for the black card. Reaction time was measured. A lower score implied better performance.

The reaction time in the working memory test was assessed by the One Back Task, and working memory was assessed by the Two Back Task. In the One Back Task, the participants were asked, using buttons "yes" or "no", to answer whether the present card was the same as the previous card. In the Two Back Task, the participants were asked (using buttons "yes" or "no") to answer whether a present card was the same as the card presented two cards ago. A lower score in speed and a higher score in accuracy indicated better performance.

Paired-associate learning was assessed by the Continuous Paired Associate Learning Task. The test had two parts. First, the participants were asked to learn and remember abstract pictures and hidden patterns in different locations. Second, the participants were requested to recall where the re-displayed hidden picture had been located. The test score was the errors during all performance sequences. Therefore, a lower score indicated better performance.

A Composite cognition score was calculated by taking the average of the standardized scores across all CogState tests. For the tests for which a lower score indicated better performance, the score was reversed (multiplied by -1) so that all outcome variables were in a uniform direction. Thus, a higher score indicated better performance.
